# Nanorod Self-Assembly in High J_c_ YBa_2_Cu_3_O_7−x_ Films with Ru-Based Double Perovskites

**DOI:** 10.3390/ma4112042

**Published:** 2011-11-17

**Authors:** Terry G. Holesinger, Matthew D. Feldmann, Boris Maiorov, Leonardo Civale, John A. Kennison, Yates J. Coulter, Paul D. Dowden, Javier F. Baca, Paul H. Tobash, Eric D. Bauer, Kenneth R. Marken

**Affiliations:** 1 Superconductivity Technology Center, 30 Bikini Atoll Rd, Los Alamos, NM 87545, USA; E-Mails: dmfeldmann@gmail.com (M.D.F.); maiorov@lanl.gov (B.M.); lcivale@lanl.gov (L.C.); jkennison@lanl.gov (J.A.K.); jycoulter@lanl.gov (Y.J.C.); pdowden@lanl.gov (P.D.D.); f.javier.baca@gmail.com (J.F.B.); kmarken@lanl.gov (K.R.M.); 2 Condensed Matter and Magnet Science, Materials Physics and Applications Division, Los Alamos National Laboratory, Los Alamos, NM 87545, USA; E-Mails: ptobash@lanl.gov (P.H.T.); edbauer@lanl.gov (E.D.B.)

**Keywords:** superconductivity, film, pulsed laser deposition, self-assembly, TEM, STEM

## Abstract

Many second phase additions to YBa_2_Cu_3_O_7−x_ (YBCO) films, in particular those that self-assemble into aligned nanorod and nanoparticle structures, enhance performance in self and applied fields. Of particular interest for additions are Ba-containing perovskites that are compatible with YBCO. In this report, we discuss the addition of Ba_2_YRuO_6_ to bulk and thick-film YBCO. Sub-micron, randomly oriented particles of this phase were found to form around grain boundaries and within YBCO grains in bulk sintered pellets. Within the limits of EDS, no Ru substitution into the YBCO was observed. Thick YBCO films were grown by pulsed laser deposition from a target consisting of YBa_2_Cu_3_O_y_ with 5 and 2.5 mole percent additions of Ba_2_YRuO_6_ and Y_2_O_3_, respectively. Films with enhanced in-field performance contained aligned, self-assembled Ba_2_YRuO_6_ nanorods and strained Y_2_O_3_ nanoparticle layers. A 0.9 µm thick film was found to have a self-field critical current density (*J_c_*) of 5.1 MA/cm^2^ with minimum *J_c_*(Θ, H=1T) of 0.75 MA/cm^2^. Conversely, *J_c_* characteristics were similar to YBCO films without additions when these secondary phases formed as large, disordered phases within the film. A 2.3 µm thick film with such a distribution of secondary phases was found to have reduced self-field *J_c_* values of 3.4 MA/cm^2^ at 75.5 K and *J_c_*(min, Θ, 1T) of 0.4 MA/cm^2^.

## 1. Introduction

Optimizing the film deposition for high-performance thick films of the high-temperature superconductor (HTS) YBa_2_Cu_3_O_y_ (YBCO) and rare-earth substituted polymorphs (REBCO) is a critical area of HTS wire development. HTS coated conductor wires are engineered composites consisting of epitaxially deposited buffer and HTS layers on bi-axially textured templates [1,2,3,4,5]. Applications of HTS wires in transmission lines, motors, and generators require operation of the conductor in moderate to high magnetic fields [6,7]. Efforts to minimize dissipation arising from vortex motion in applied fields have resulted in HTS films that are highly-engineered materials consisting of YBCO or REBCO films with well-defined distributions of nano-sized defects and/or second-phases. These defects and secondary phases need to have dimensions on the order of 5–10 nm in order to effectively pin vortices and improve the overall performance in self and applied magnetic fields at liquid nitrogen temperatures and below [8].

Maximizing the temperature (T) and magnetic field (**H**) dependent critical current density (*J_c_*) requires optimization of the size, density and distribution of nanoscale structures which act as vortex pins [9,10]. The anisotropic nature of YBCO and its effects on *J_c_*(H, Θ, T) further complicate this process by requiring optimization of pinning defects for different orientations of applied field [11]. Significant effort has been spent to introduce nanoscale pinning structures in materials such as YBCO and REBCO though the incorporation of second phase materials such as BaZrO_3_ [12,13,14], BaSnO_3_ [15], Y_2_O_3_ [16] Y_2_BaCuO_5_ (Y-211) [17] or rare-earth tantalates [18] to the YBCO source material to induce second phase growth. Efforts are made to maximize self-field *J_c_*, while maintaining the highest possible minimum *J_c_* for any applied field orientation (Θ) [14]. Recent results combining both Y_2_O_3_ and BaZrO_3_ additions in a single-layer, 1.95 μm thick film returned a *J_c_*(75.6K, self-field) of 5.2 MA/cm^2^ (1010 A/cm-w) and a minimum *J_c_*(75.6K, 1T) of 1.2 MA/cm^2^ (240 A/cm-w) in the maximum Lorentz force measurement configuration [19]. Self-assembled Y_2_O_3_ nanoparticle layers and Zr-based nanorods were produced in these films as pinning defect structures for **H**||ab and **H**||c, respectively.

Perovskite-based additions have garnered the most interest as a class of materials for incorporation into REBCO films. It was suggested early on that perovskites could be easily incorporated into YBCO or used as substrates due to their similar crystal structures to YBCO [20,21,22,23,24]. Many Ba-containing perovskites, including those with Zr [25], are double-perovskites of the general form A_2_BB′O_6_. One early work in the field reported the formation of Ba_2_YNbO_6_ within YBCO films, but without improvements in performance [24]. However, renewed interest in the double perovskites was recently generated from works with pulsed laser deposition (PLD) films in which BaNbO_3_ additions to an ErBa_2_Cu_3_O_y_ source material resulted in self-assembled nanorods of the double perovskite Ba_2_ErNbO_6_ within the Er-123 film matrix [26,27]. The nanorods were shown to easily span the entire thickness of the ErBa_2_Cu_3_O_y_ film. Other groups quickly followed with positive results using Ba_2_YNbO_y_ additions to Y-123 films [28,29,30]. Recently, extremely high pinning forces (*F_p_*) were obtained in a 0.5 μm thick film with *J_c_*(75.6 K, 1T, H||*c*) > 2 MA/cm^2^ and *F_p_* (H||c) values in excess of 30 and 120 GN/m^3^ at 75.5 K and 65 K, respectively [28,29,30]. Such results have spurred further efforts in exploring other possible perovskite-based additions. In this work, we report our results on the incorporation of a Ru-based double-perovskite phase into bulk YBCO and the deposition of YBCO films with Ba_2_YRuO_y_ nanorod structures and enhanced *J_c_* performance in self and applied magnetic fields.

## 2. Results and Discussion

It is well known that a large number of perovskites can be formed with Ba [31,32,33] However, their incorporation into YBCO bulk and film forms must occur with minimal detrimental effects to the superconducting phase. Ruthenium was investigated as a potential material for addition to YBCO based on its known formation into the ordered, tilted perovskite A_2_BB’O_6_ of composition Ba_2_YRuO_6_ [32]. Its compatibility with YBCO was tested with mixtures of bulk YBCO and RuO_2_ additions and its potential for performance improvements though its incorporation as nanorod secondary phases in YBCO films. It should be noted that Ru is an expensive material. Based on 5 mol % additions of Ba_2_YRuO_6_ to YBCO in the fabrication of the targets used for PLD, costs for the HTS materials would increase by 13 to 22%, depending on the starting purity of the RuO_2_.

### 2.1. Ba_2_YRuO_6_ Double-Perovskite

**Figure 1:**
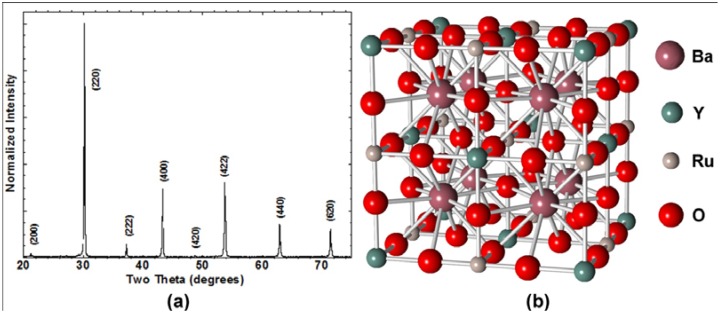
(**a**) XRD powder diffraction scan of Ba_2_YRuO_6_. The refined lattice parameter from this material was found to be *a_0_* = 8.3381(4); (**b**) Crystral structure of Ba_2_YRuO_6_ based on space group *Fm-3m* and atom positions from reference [32].

Ba_2_YRuO_6_ was synthesized as a single phase material using conventional milling and sintering processes. Shown in Figure 1 is the XRD scan of the single-phase material and its ordered crystal structure. Table 1 contains the XRD data pertaining to observed and calculated peak positions and intensities. A single-phase diffraction pattern characteristic of a perovskite was obtained. The refined lattice parameter based on the integrated Bragg reflections obtained from diffraction experiments using Si (NIST SRM 640a) mixed in with the sample was *a_0_* = 8.3381(4). Observed intensities and peak positions are consistent with previous reported work on this phase which includes ordering of the Y and Ru atoms on the B sites of the perovskite structure [32].

**Table 1:** Table of observed and calculated XRD characteristics for Ba_2_YRuO_6_. Calculated values are based on the earlier work of Battle *et al.* with *a_0_* = 8.3390(5) and space group *Fm-3m*.

h	k	l	2Q(obs)	Int(obs)	d(obs)	2Q(calc)	d*(calc)	Int*(calc)
1	1	1	18.415	1	4.814	18.4549	4.80344	0.8
0	0	2	21.282	1.3	4.1715	21.341	4.1599	0.6
0	2	2	30.283	100	2.949	30.3605	2.94149	100
1	1	3				35.7636	2.50851	0.1
2	2	2	37.323	5.1	2.4073	37.4115	2.40172	5.8
0	0	4	43.362	29	2.085	43.4709	2.07995	30.9
1	3	3				47.6004	1.90869	0
0	2	4	48.753	0.5	1.8663	48.9167	1.86036	0.5
2	2	4	53.805	31.8	1.7024	53.9433	1.69827	39.3
1	1	5				57.5095	1.60115	0.2
0	4	4	63.005	13.8	1.4742	63.1634	1.47075	18.7
1	3	5				66.4205	1.4063	0.1
0	0	6				67.4878	1.38663	0.3
0	2	6	71.504	12	1.3184	71.6808	1.31548	17.4
3	3	5				74.7588	1.26876	0
2	2	6				75.7744	1.25426	1.5
4	4	4	79.604	3.5	1.2033	79.7949	1.20086	6.2
1	1	7				82.7762	1.16501	0.1
0	4	6				83.7652	1.15375	0.2
2	4	6	87.485	11.3	1.1141	87.7056	1.11178	21.4

### 2.2. Bulk YBCO with RuO_2_ Additions

**Figure 2:**
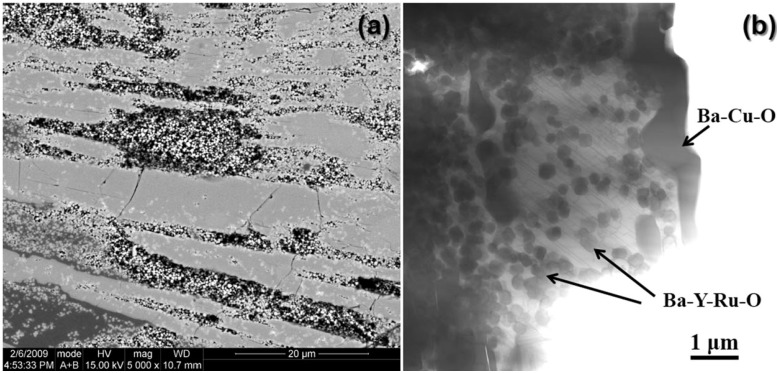
(**a**) SEM backscattered electron image and (**b**) STEM Z-contrast image showing the large amount of submicron Ru-containing secondary phases formed in the bulk YBCO sample containing 9 wt % additions of RuO_2_. The inclusion of round, sub-micron grains and clusters of grains within the YBCO phase is readily apparent in (b). Ba-Cu-O is also present in the form of large irregularly shaped phases as indicated.

**Figure 3:**
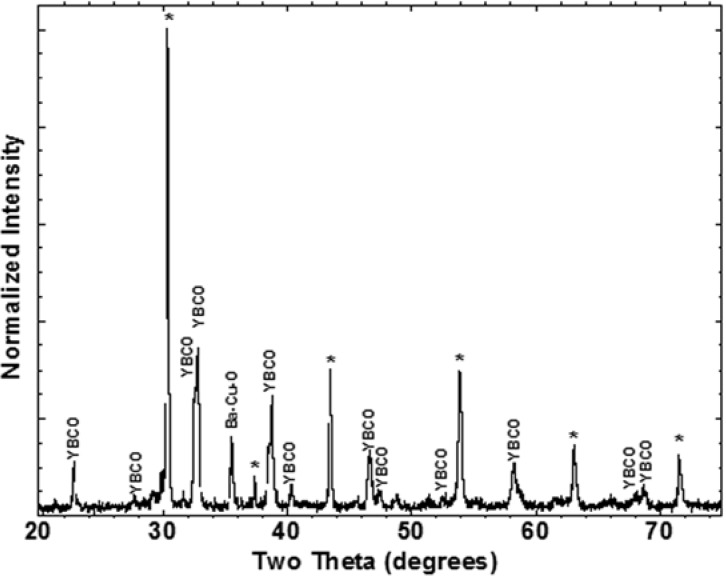
XRD scan of the bulk sample consisting of YBCO powder with 9 wt % RuO_2_ additions. Most peaks in the XRD scan can be indexed to YBCO or Ba_2_YRuO_6_ (*).

Shown in Figure 2 are SEM and STEM images of material taken from a pressed pellet containing 9 wt % RuO_2_ additions to stoichiometric YBCO powder. The resulting material was multiphase containing primarily YBCO and a sub-micron sized secondary phase containing Ru along with small amounts of BaCu_2_O_y_ and CuO, STEM/EDS analysis suggested an approximate ratio of 2:1:1 of Ba:Y:Ru with a small amount of Cu present in the material. However, the copper content in this phase was not consistent with the reported formation of the 2411 phase in other works [34]. Within the limits of EDS, no Ru was detected within the YBCO phase. Figure 3 contains the XRD analysis of the pellet which showed a multiphase material with the major peaks identified as either YBCO or the double-perovskite Ba_2_YRuO_6_. The intensities of the peaks associated with Ba_2_YRuO_6_ are consistent with the observed amounts of the double-perovskite phase present in the sample.

### 2.3. YBCO with Self-Assembled Nanorods of Ba_2_YRuO_y_

Films containing the Ba_2_YRuO_y_ (BRYO) secondary phase were grown from PLD targets consisting of YBa_2_Cu_3_O_y_ with 2.5 mol % additions of Y_2_O_3_ and 5 mol % additions of Ba_2_YRuO_6_. The addition of the extra Y_2_O_3_ was included for the purposes of producing the two-phase pinning mesostructure of strained Y_2_O_3_ nanoparticle layers and perovskite-based nanorods [19]. High self-field *J_c_* values of 5.0, 5.1, 4.5, and 3.4 MA/cm^2^ were obtained in measurements at 75.5 K and self-field for films of thickness 0.85, 0.9, 0.95 and 2.3 μm, respectively.

**Figure 4:**
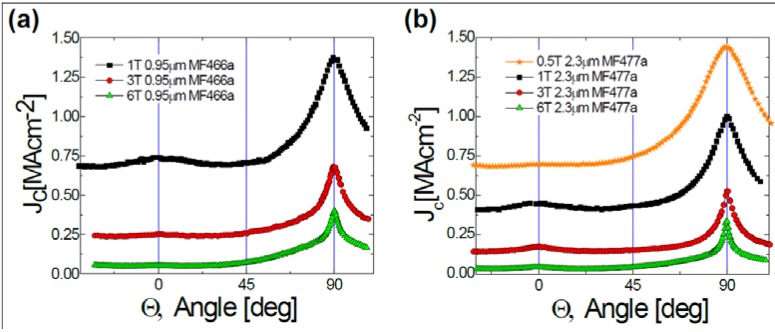
(**a**) Measurement of the *J_c_* characteristics at 75.5 K as a function of magnetic field strength and applied field orientation for a 0.95 μm thick film; and (**b**) a 2.3 μm thick film.

Detailed *J_c_* characteristics of the 0.95 and 2.3 µm thick films are shown in Figure 4, which contains the angular dependence of *J_c_* for applied magnetic fields of 1, 3, and 5 T. The 0.95 µm film had a self-field *J_c_* value of 4.5 MA/cm^2^ along with *J_c_*(H=1T, H||c) = 0.75 MA/cm^2^ and *J_c_*min(Θ, H=1T) = 0.7 MA/cm^2^. The 2.3 µm thick film had a self-field *J_c_* value of 3.4 MA/cm^2^ along with *J_c_*(H=1T, H||c) = 0.45 MA/cm^2^ and *J_c_*min(Θ, H=1T) = 0.4 MA/cm^2^. For reference, a plain YBCO without nanorod structures would have a *J_c_*min(Θ, H=1T) = 0.4 MA/cm^2^ [35]. While there was clearly an *J_c_*(H) improvement for the 0.95 micron thick film, there was essentially none above what would be expected for plain YBCO for the 2.3 micron thick film. Hence, a close comparison of the microstructural properties with *J_c_* performance is necessary for understanding these differences.

**Figure 5:**
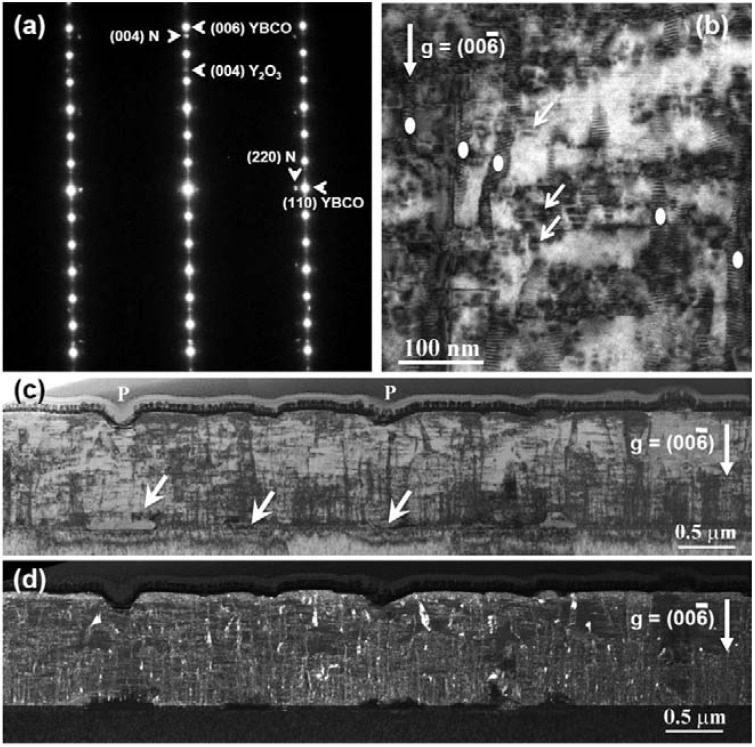
(**a**) TEM diffraction data for the 0.9 µm thick film showing the alignment of the Ba_2_YRuO_6_ phase with YBCO; (**b**) High magnification bright field TEM image showing the BRYO nanords (white dots) and strained and tilted Y_2_O_3_ (arrows) nanoparticle layers. Low magnification TEM bright field (**c**) and weak-beam (**d**) images showing a mostly uniform distribution of secondary phases in this particular TEM specimen.

**Figure 6:**
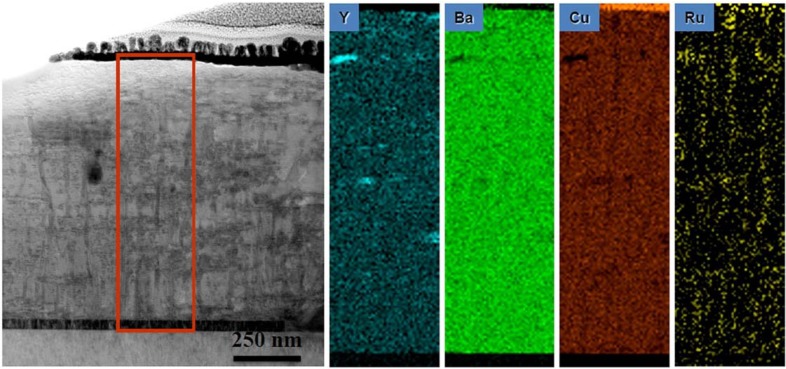
STEM bright-field image and associated energy dispersive spectral imaging showing Ru in secondary phases.

**Figure 7:**
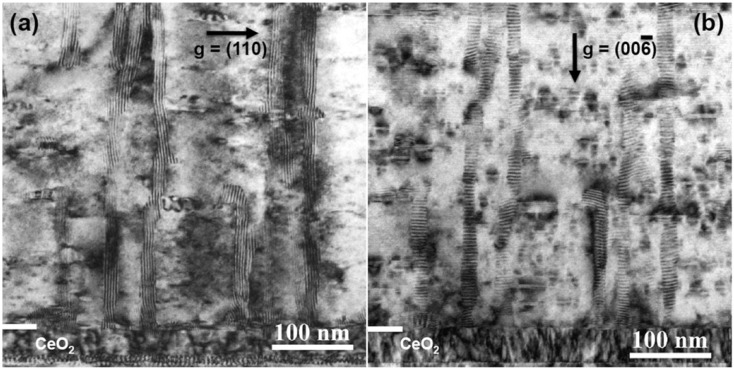
(**a**) TEM two-beam, bright field image of the nanorods imaged using conditions g = (110) and (**b**) g = (00–6). The continuous Moiré fringe structure in (**a**) shows that each nanorod is a single phase rather than a stack of individual particles.

Figure 5 shows the general microstructure of a 0.9 µm thick film which had a self-field *J_c_* of 5.1 MA/cm^2^. This film also showed enhanced performance with *J_c_*(H=1T, H||c) = 0.8 MA/cm^2^ and *J_c_*min(Θ, H=1T) = 0.75 MA/cm^2^. The second phase assemblage or mesostructure within the film consisted of BRYO nanorods aligned with the film normal and tilted Y_2_O_3_ nanoparticle layers. The STEM and EDS spectral images of Figure 6 show the Ru to be associated with the BYRO nanorod structures that are continuous though the full thickness of the film. EDS analysis of the BYRO nanorods showed a small copper content. However, the amount detected was not consistent with the reported 2411 phase [36]. The continuous nature of these nanorod structures is confirmed with the Moire fringe analysis shown in Figure 7. Growth of the nanorods starts at the CeO_2_ buffer layer. Indexing of the diffraction pattern the double perovskite in Figure 5(a) revealed the nanorod phases to have an alignment defined by [1,2,3,4,5,6,7,8,9,10] BRYO || [1,2,3,4,5,6,7,8,9,10] YBCO with (220) BRYO || (110) YBCO, identical to the one found for Ba_2_YNbO_6_ nanorods [28]. The lobe-shaped contrast around the Y_2_O_3_ particles in the TEM image (arrows Figure 5(b)) indicates that the alignment of the Y_2_O_3_ particles with the YBCO is highly strained. Hence, these additions were nearly successful in the reproduction of the idealized, two phase, strained nanoparticle layer and nanorod pinning mesostructure as first reported by the Los Alamos group [14,19]. The only difference between the ideal structure and the mesostructure observed here being the continuous nature of the BYRO nanorods *versus* the short, segmented structure of the Zr-based nanorods.

The improvements in observed performance, relative to plain YBCO are then understood quite well. In this particular TEM FIB specimen, the distribution of the secondary phases was very uniform as indicated by the low magnification images of Figure 5(c,d). Figure 5(d), the dark-field, weak-beam TEM image shows this distribution quite well. Weak-beam diffraction contrast is well-suited for imaging the nano-sized phases and structures at relatively low magnifications as the strain associated with the phases allows the phases to be highlighted in the images. The arrows in Figure 5(c) indicate localized reactions between the substrate and CeO_2_ buffer layer to form BaCeO_3_ [37]. This latter phase is not aligned and appears dark against the contrast from the YBCO film in the weak-beam image of Figure 5(d). The low-magnification TEM images also show the surface porosity “P” that is commonly found in these PLD films when the surface morphology is examined in the SEM. While the porosity depth is small for these nominally 1 micron thick samples, it becomes more pronounced for the thicker films as shown below.

**Figure 8:**
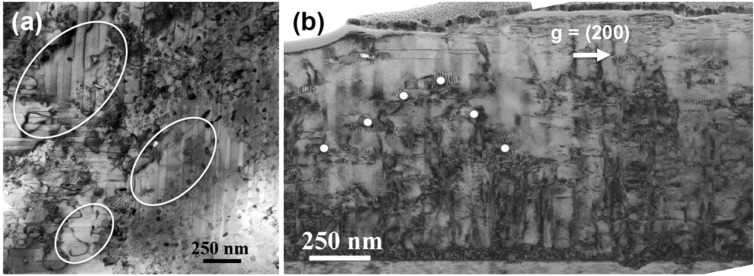
(**a**) Areas devoid of secondary phases are highlighted in the plan-view, bright-field STEM image of the 0.95 mm thick film taken from an area near the top surface of the film. (**b**) White dots in the cross-sectional TEM bright-field mark large Y_2_O_3_ particles that cap or disrupt the growth of the BRYO nanorods, resulting in nearby areas of the YBCO film to be devoid of secondary phases.

While an enhancement in the in-field performance of the films of thickness 0.85 to 0.95 µm in thickness was observed, the absolute level of performance was still not to the level of the best, optimized YBCO films with BaZrO_3_ and Y_2_O_3_ additions. Hence, further microstructural investigations were undertaken using plan view (S)TEM. Figure 8(a) shows a STEM plan view image where the observed material is located near the top surface of the 0.9 µm thick film described above. As indicated by the circles, areas could be identified that were devoid of any secondary phases. Further investigations of this film with additional cross-sectional specimens showed that indeed, these regions existed within the films. These clear areas were found to occur above regions where interruptions occurred in the development of the nanorod/strained nanoparticle mesostructure. As shown in the example of Figure 8(b), growth of the nanorod/nanoparticle mesostructure did not occur across the full thickness of the film. Instead, there was a change to a structure that ended with large Y_2_O_3_/ Ba_2_YRuO_6_ composite particles capping the growth of the nanorods. Above this structure, a region free of secondary phases could be found. This degradation of the mesostructure was present in moderate amounts in the submicron films and would explain why the in-field performance of these first films were not to the levels found in related films that have been optimized for additions of BaZrO_3_, for example [19]. The mechanism for this mesostructure degradation will be the focus of future work.

The identification of the mesostructure degradation in regions of the nominally 1 µm thick films was important as it leads to the basis of understanding performance degradation in the 2.3 µm thick film. While the self-field *J_c_* value of 3.4 MA/cm^2^ obtained in the latter film was very respectable, its *J_c_* performance in field was no different from that measured in plain YBCO films without c-axis aligned defects. Now it has been commonly observed in many works that *J_c_* decreases as a function of film thickness (See reference [9] and works cited therein). It is tempting to assume that this is an intrinsic property of these thick films. However, in recent works where detailed microstructural analysis was coupled to film performance in high quality PLD or ex situ metal orgranic deposition (MOD) films, it was shown that *J_c_* becomes independent of thickness when the microstural features are uniform though thickness [10,19,38]. The 2 micron, 5 MA/cm^2^ (75K, SF) film reported by Feldmann *et al.* is a clear illustration of the effects of uniformity though thickness and its pronounced influence on film properties. It also strongly supports the notion that the *J_c_* dependence for film thickness is an extrinsic property.

**Figure 9:**
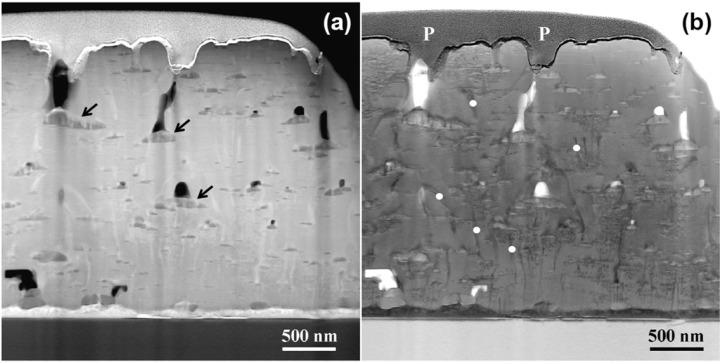
(**a**) STEM Z-contrast image showing a distribution of large Y_2_O_3_ particles, many of which appear to have a composite structure containing a second, higher Z material (arrows); (**b**) Bright field STEM image showing the splayed, ill-formed, and non-continuous network of BRYO nanorods (white dots) within this thick film. Surface pores, which extend into the sample for appreciable distances in this thick film, are marked with a “P”.

**Figure 10:**
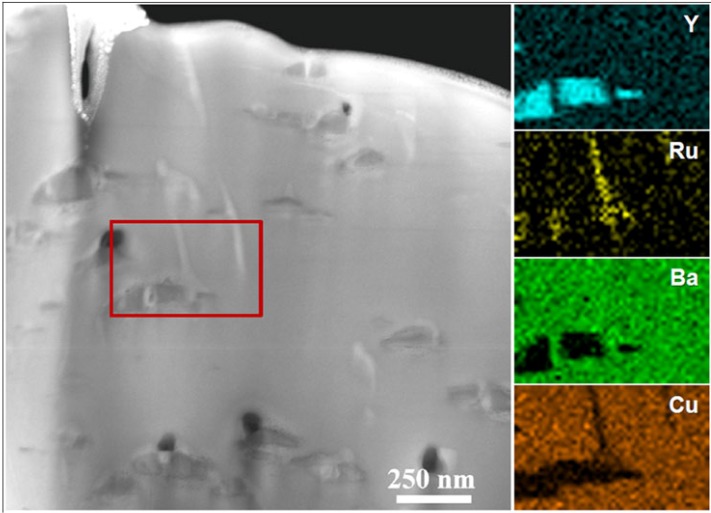
STEM Z-contrast image and associated EDS spectral images showing that large, striped secondary phases are actually composites of Y_2_O_3_ and the BRYO double-perovskite.

The STEM images of Figure 9 show significant degradation of the film structure, especially for those sections of the film more than 0.5 microns from the CeO_2_ buffer. The z-contrast image, Figure 9a, shows a distribution of large secondary phases that appear to be composites of multiple phases based on their z-contrast. The spectral imaging data of Figure 10 revealed these composite phases to be composed of Y_2_O_3_ and Ba_2_YRuO_6_. Hence, we attribute the degradation of the in-field properties to the consolidation of the nanorod/strained nanoparticle mesostructure into large composite secondary phases. The remaining BYRO nanorods that are still present in this sample are much larger and irregularly shaped compared to those found in the nominally 1 µm thick films. Understanding the underlying causes to the changes in this mesostructure is important for incorporating these structures into continuous processing of YBCO films for long-length wires [39,40]. Related work by one of the authors suggests that these same degradation mechanisms can also occur in films with BaZrO_3_ or Ba_2_YNbO_6_ additions [41]. The difference in the mesostructure would also explain the early results with Ba_2_YNbO_6_ additions in which the phases were present, but no appreciable increase in performance was observed [24].

## 3. Experimental Section

Ba_2_YRuO_6_ was prepared by mixing individual elemental oxide precursors and milling for 8 h in a Retsch mortar grinder with distilled H_2_O, Isopropanol, and KD-1 dispersant. After milling, the slurry was dried on a Schlink line. The dried powder was hand-ground with a mortar and pestle and pressed at 5 K psi into 1.25 cm diameter pellets. The pellets were heat treated in flowing O_2_ in a high-temperature box furnace. The pellets were ramped to 1000 °C at 10 °C/h and held for 15 h. They were then ramped at 1 °C/h to 1550 °C and held for 15 h. The pellets were then cooled to 1000 °C at 2 °C/h for a final dwell of 2 h, and then ramped to room temperature at 15 °C/h. Bulk YBa_2_Cu_3_O_7_ (YBCO) pellets with additions of 9 wt % RuO_2_ were prepared by milling stoichiometric mixtures of prepared YBCO with RuO_2_. The powders were pressed into 1.25 cm diameter pellets which were then sintered at 1000 °C for 50 h, followed by an annealing step at 400 °C for 50 h in flowing oxygen. Powder X-ray diffraction (XRD) patterns were obtained on a Scintag X-1 Advanced Diffraction System using filtered Cu Ka radiation (λ = 1.5406 Å). XRD patterns were taken with and without a Si reference material (NIST SRM 640a); scans with Si were used for quantitative structure refinements. Scans were collected at room temperature in Θ-Θ mode (2Θ = 90°) with a step size of 0.02 and collection time of 10 sec/step. The XRD data collected was analyzed though the Jade 6.5 software package which as used for phase identification and determination of refined unit cell parameters [42]. Crystal structures and calculated diffraction patterns were obtained using CrystalMaker^®^ [43]. Targets for pulsed laser deposition (PLD) were made by mixing precursor powders in the desired ratios, followed by milling in isopropanol in a Retsch motar grinder for 8 h. Powders were dried on a shlenk line and then calcined as a loose powder for 15 h at 900 °C. This powder was then lightly ground in a glove box, pressed into a 5.5 cm diameter target 0.5 cm thick, and then sintered at 925 °C and 950 °C for 15 h each with a furnace cool to 400 °C for 5 h in between steps. The sintering process ended with a low temperature anneal at 400 °C for 50 h.

Films of thickness 0.8 to 2.3 µm were grown by PLD using a KrF laser (248 nm). The target material was YBa_2_Cu_3_O_7_ (YBCO) with 5 mol % additions of Ba_2_YRuO_6_ and 2.5 mol % additions of Y_2_O_3_. Substrates were single crystals of either SrTiO_3_ or Y-stabilized Zr_2_O_3_ with a 40 nm thick CeO_2_ buffer layer. The deposition temperature during growth was either 775 °C or 795 °C. Films were patterned for transport measurement using standard photolithography and wet etching techniques. Before photolithography, Ag pads were deposited by thermal evaporation and the films subsequently annealed at 500 °C for 30 minutes in flowing O_2_. Values of critical temperature (*T_c_*) were determined by an inductive method and values of *J_c_* were determined using the standard 1 µV/cm electric field criterion and a four-point transport measurement. All bridges were 1.5 mm long and approximately 200 µm wide. Measurements of *J_c_* as a function of field orientation were performed in the maximum Lorentz force configuration, with Θ being the angle between the film normal and field vector. *J_c_* measurements in applied magnetic fields were performed in a 1 T split coil electromagnet or in a 7 T split coil superconducting magnet. (Scanning) transmission electron microscopy ((S)TEM) characterizations were performed on foils thinned by either conventional dimple-polishing and ion-milling or foils prepared with a focused ion beam (FIB) technique.

## 4. Conclusions

We have shown that the double-perovskite Ba_2_YRuO_6_ can be an effective and compatible addition for both bulk and thick film YBCO materials. In the former case, this secondary phase distributed itself within the YBCO material as small, sub-micron randomly-oriented secondary phases. Additions of 5 mol % Ba_2_YRuO_6_ and 2.5 mol % Y_2_O_3_ to YBCO thick films resulted in mesostructures within the film of aligned, continuous BYRO nanorod and strained Y_2_O_3_ nanoparticle structures. The departure from the idealized mesostructure being the presence of the continuous, as opposed to the optimal short segmented, nanorods. Self-field *J_c_* values up to 5.2 MA/cm^2^ were obtained in 0.95 µm thick films with improved in-field performance as indicated by the measured minimum *J_c_*(Θ, H=1T) of 0.75 MA/cm^2^ We also showed how the degradation to the mesostructure can lead to performance that is not very different from YBCO films without additions. High self-field *J_c_* values are still possible as indicated by the measurement of 3.4 MA/cm^2^ (self-field, 75.5 K) in a 2.3 µm thick film with the BYRO and Y_2_O_3_ additions. However, the degradation of the mesostructure within this thick film results in a measured minimum *J_c_*(Θ, H=1T) of only 0.4 MA/cm^2^. The observed mesostructure degradation is important in understanding the variable range of performance one can obtain when investigating pinning additions to YBCO films. It is believed that continued optimization of the deposition process and chemistry for Ba_2_YRuO_6_ and Y_2_O_3_ additions would lead to performance levels comparable to the best achieved with BaZrO_3_ and Y_2_O_3_ additions.
